# Differential Evolution Based IDWNN Controller for Fault Ride-Through of Grid-Connected Doubly Fed Induction Wind Generators

**DOI:** 10.1155/2015/746017

**Published:** 2015-10-01

**Authors:** N. Manonmani, V. Subbiah, L. Sivakumar

**Affiliations:** ^1^Department of EEE, Sri Krishna College of Engineering and Technology, Coimbatore 641008, India; ^2^Department of EEE, PSG College of Technology, Coimbatore 641004, India; ^3^Sri Krishna College of Engineering and Technology, Coimbatore 641008, India

## Abstract

The key objective of wind turbine development is to ensure that output power is continuously increased. It is authenticated that wind turbines (WTs) supply the necessary reactive power to the grid at the time of fault and after fault to aid the flowing grid voltage. At this juncture, this paper introduces a novel heuristic based controller module employing differential evolution and neural network architecture to improve the low-voltage ride-through rate of grid-connected wind turbines, which are connected along with doubly fed induction generators (DFIGs). The traditional crowbar-based systems were basically applied to secure the rotor-side converter during the occurrence of grid faults. This traditional controller is found not to satisfy the desired requirement, since DFIG during the connection of crowbar acts like a squirrel cage module and absorbs the reactive power from the grid. This limitation is taken care of in this paper by introducing heuristic controllers that remove the usage of crowbar and ensure that wind turbines supply necessary reactive power to the grid during faults. The controller is designed in this paper to enhance the DFIG converter during the grid fault and this controller takes care of the ride-through fault without employing any other hardware modules. The paper introduces a double wavelet neural network controller which is appropriately tuned employing differential evolution. To validate the proposed controller module, a case study of wind farm with 1.5 MW wind turbines connected to a 25 kV distribution system exporting power to a 120 kV grid through a 30 km 25 kV feeder is carried out by simulation.

## 1. Introduction

For the past few years, doubly fed induction generators (DFIGs) occupied the world's largest share of wind turbines as a variant to traditional variable speed generators. The designed system should be in a position to operate over a wide range of wind speeds for achieving optimal efficiency in order to follow the optimal tip-speed value. Henceforth, the generator's rotor is to be designed to operate in a variable rotational speed. DFIGs are therefore designed to operate under both super- and subsynchronous modes with a speed range of the rotor being in accordance with that of the synchronous speed. The stator circuit module is connected directly to that of the grid; on the other hand the windings of the rotor are connected through slip rings to a three-phase converter. In case of a variable speed system, when the speed range requirements are low the DFIG provides satisfactory performance and this is found to be enough for the speed range employed with the wind power. In DFIG module, an AC-to-AC converter is incorporated in the induction generator rotor circuit and the converters are to be rated to handle a fraction of the total power. The total power is the rotor power which forms around 32% of nominal generator power. As a result, the occurrences of losses in the converter module can be reduced in comparison with that of the system where the converter is to handle the complete power, and the entire cost is minimal because of the partial rating of the power electronic circuits. This section details the earlier studies and simulations carried out in the applicability and design of DFIGs in wind turbines.

Vieira et al. [1] carried out a work on genetic algorithm based optimal controllers to the rotor-side converter of doubly fed induction generators (DFIGs), the DFIG converter control action performed by proportional and integral controllers. The presented approach improves transient stability margin of the power system and also has better global system dynamic behavior during and after the fault period. In [2] Nagaria et al. analyzed various control strategies for frequency support in DFIG-based WECS; based on the comparative analysis the following point can observe minimum frequency deviation and maximum release of energy obtained by tuning the controller parameter using PSO and improved performance is achieved by inertial response of the system with the PSO optimized proportional controller. Bhatt et al. [3] presented doubly fed induction generator dynamic participation in automatic generation control; frequency response of the system is improved by frequency control support function and craziness-based particle swarm optimization (CRPSO) utilized to obtain the optimal DFIG parameter, transient response of area frequency, and tie-line power deviation.

A novel robust controller [4] is suggested in a stationary reference frame for grid-integrated doubly fed induction generators based wind turbines. The voltage and flux equations in *αβ* coordinates used DFIG dynamic model design; the disturbances and uncertainties intrinsic to the system are accounted for as perturbation terms are included to the nominal model. ANN [5] based adaptive PI controllers on DFIG driven based wind turbine in order to improve transient performance of DFIG in all wind speed conditions. ANN is used to predict the optimal values of parameter and adaptive PI controllers according to different wind speeds dynamically change PI gain values. A genetic algorithm [6] based PID controller coefficients adjustment on wind energy conversion system with doubly fed induction generator. Liu et al. [7] pointed out the DFIG speed controller based on the nonlinear intelligent model predictive control algorithm. SVM is adapted to establish the speed of DFIG and PSO is utilized to solve the rolling optimization; the forecasted error is used to form a closed-loop control. Various computational intelligence based controls of wind turbine generators such as PSO, MVO, adaptive critic design based adaptive control, and shunt FACTS devices are analyzed in [8].

Multiobjective optimization [9] control methodology of a doubly fed induction generator (DFIG) considering the distorted grid voltage conditions was dealt with. The four various control targets are suggested to restrain the harmonic components in stator/rotor current or the ripples in the stator output active/reactive power and electromagnetic torque. Vinothkumar and Selvan [10] carried out a work on a new enhancement of fault ride-through capability and subsequent betterment of rotor speed stability of doubly fed induction generator based wind farms. The wind turbine mechanical energy input is stored during the grid fault and at the moment of fault clearance it is utilized; hence the charging DC-link capacitor by grid-side converter is relieved. Particle swarm optimization [11] based optimal VAR control in grid-connected DFIG-based wind farm has been presented to optimize reactive power from reactive power capability (RPC) of DFIGs in order to obtain system active power loss minimization.

A hybrid particle swarm optimization [12] (HPSO) algorithm for the DFIG optimum design is presented in this work and the searching performance is improved by adapting the diversity-guided adaptive mutation and fitness-guided individual fuzzy inertia weight. An optimal grid-integrated-to-the-rotor type doubly fed induction generator design for wind turbine systems is developed in [13]. Kriging model based on Latin hypercube sampling and genetic algorithm are adapted to maintain the efficiency and maximize the torque per weight. Amelian et al. [14] carried out a work on a small signal stability improvement based on eigenvalue analysis of a wind turbine-based doubly fed induction generator in a micro grid environment. The stability margin and damping ratio of critical modes are enhanced by using particle swarm optimization algorithm.

An optimal VAR expansion based on capability curve of DFIG wind farm has been proposed in [15]. The proposed approach reduces the sum of the annual investment cost of the new VAR devices and the annual expected energy loss cost. Design of adaptive controller [16] using optimal fuzzy logic of doubly fed induction generator (DFIG) wind turbine for standalone power system frequency control has been proposed in this work. The particle swarm optimization is employed to adapt and optimize the fuzzy logic design membership function and the control rules.

Tang et al. [17] carried out a work on an optimal control of doubly fed induction generators- (DFIGs-) based wind generation adapting trajectory and frequency domain information based sensitivity analysis and particle swarm optimization. The critical parameters, the unified dominate control parameters (UDCP), are identified using sensitivity analysis and PSO is utilized to obtain the control goal by searching the optimal values. A fuzzy controller is proposed in [18], based on fuzzy adaptive theta particle swarm optimization (FAΘPSO) applied to a doubly fed induction generator (DFIG) to run at medium voltage; FAΘPSO algorithm is used to obtain optimum values of membership functions. A load frequency control (LFC) of micro grids connected with main grids in a regulated and deregulated environment; the PSO (particle swarm optimization) tuned PI controller gains have improved performance compared to the conventional PI controller parameters proposed in [19]. An adaptive neurofuzzy inference system [20] controller is pointed out for doubly fed induction generator based wind turbine. The presented approach controls the current and voltage ripple within 0.01 pu as well as reducing the power loss.

Kong et al. [21] proposed data-driven based modeling of doubly fed induction generator wind turbine system adapting neural networks. The DFIG model is developed based on neural networks and neurofuzzy networks by using large amount of input-output online measurement data from the selected months. An RSC controller [22] is proposed for DFIG-based wind power generation system. In this case, the stator-flux-oriented (SFO) vector control based design is adapted and RSC controller for DFIG with double closed-loop system is modeled using rotor current control loop and power control loop along with proportional integral (PI) controller. Wu et al. [23] suggested combination of particle swarm optimization (PSO) and orthogonal method for wind turbines parameter tuning of doubly fed induction generator systems. A virtual resistance control approach for high voltage ride-through [24] of doubly fed induction wind generators using particle swarm optimization is presented in this work. Zhang et al. [25] pointed out design of additional impedance-based SSR damping controller for power system with DFIG wind turbine; the additional SSR damping controller effectively suppresses the SSR.

It is to be noted that, in all the above discussed earlier studies on design and control of DFIG, the control strategies are complex in nature yielding unstable nature of the system and voltage rating of rotor is high enough in comparison with that of the megawatt rating of the wind turbine generators which possess stator to rotor winding turns ratio between 2.5 and 3. In this work, a novel control strategy employing DE based IDWNN controller is proposed which makes the DFIG-based system of high stable nature and the designed system simulation is carried out choosing appropriate turns ratio. This paper also attempts to handle the fault ride-through capability of wind turbines and to make them more stringent. The remaining part of the paper is organized as follows.  Section 2 presents the modeling of DFIG and the proposed controller design employing DE-IDWNN controller is discussed in Section 3.  Section 4 presents the applicability of the proposed DE-IDWNN for the fault ride-through of DFIG module. The simulation results and the validation of the proposed controller are performed in Section 5. The conclusions with the findings of the proposed work are summarized in Section 6.

## 2. Modeling of DFIG Wind Turbine System

**Figure 1:**
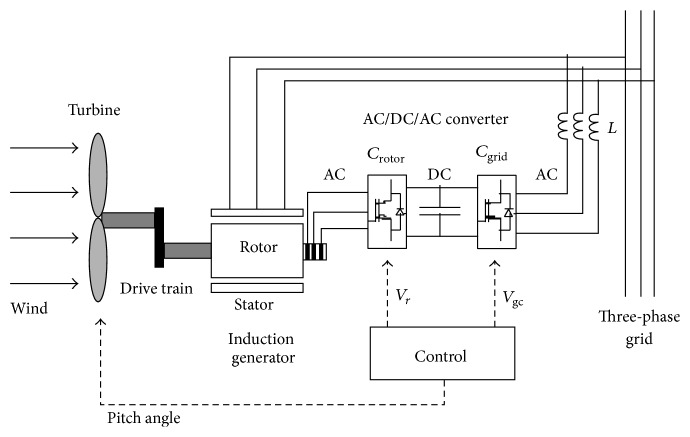
Wind turbine and the doubly fed induction generator system.

The schematic diagram of the wind turbine and DFIG is as shown in Figure 1.

The AC-DC-AC converter system of DFIG is found to be divided into two components: the rotor-side converter (*C*_ROTOR_) and the grid-side converter (*C*_GRID_). Both *C*_ROTOR_ and *C*_GRID_ are voltage-sourced converters which employ forced commutated power electronic devices (IGBTs) for synthesizing an AC voltage from a DC voltage source. On the DC side, a capacitor is connected which acts as a DC voltage source. For connecting *C*_GRID_ to the grid, a coupling inductor *L* has been used. Slip rings and brushes are used to connect the three-phase rotor winding to *C*_ROTOR_ and the three-phase stator winding is directly connected to the grid. The power acquired by the wind turbine is converted into electrical power by the induction generator and this is transmitted to the grid by the stator and the rotor windings. The control system module is employed to generate the pitch angle command and the voltage command signals *V*_*R*_ and *V*_*G*_ for *C*_ROTOR_ and *C*_GRID_, respectively. These signals tend to control the power of the wind turbine, the DC bus voltage, and the voltage or reactive power at the grid terminals.

The equations employed for computing mechanical power and the stator electric power output are as follows:

(1) $$\begin{array}{c} P_{m}=T_{m}\omega_{r}\\ P_{s}=T_{e}\omega_{s}, \end{array}$$

where *P*_*m*_ is mechanical power captured by the wind turbine and transmitted to the rotor, *P*_*s*_ is stator electrical power output, *T*_*m*_ is mechanical torque applied to rotor, *T*_*e*_ is electromagnetic torque applied to the rotor by the generator, and *ω*_*r*_ and *ω*_*s*_ are the rotational speed of rotor and of the magnetic flux in the air-gap of the generator, respectively. In case of lossless generator the mechanical equation is given by

(2) $$\begin{array}{c} J_{d\omega rdt}=T_{m}-T_{e}. \end{array}$$

**Figure 2:**
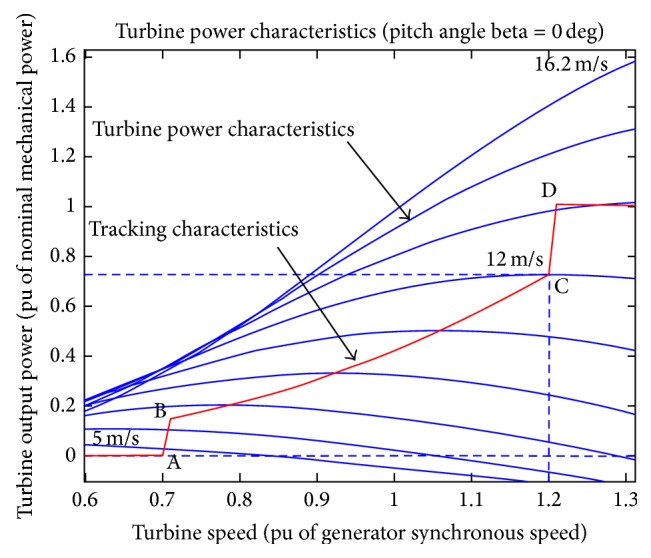
Turbine and tracking characteristics.

Considering steady state at constant speed, in case of lossless generator *T*_*m*_ = *T*_*e*_ and *P*_*m*_ = *P*_*s*_ + *P*_*r*_, where *P*_*r*_ is the rotor electrical power output and *J* is the rotor and wind turbine inertia coefficient. The rotor electrical power output is given by

(3) $$\begin{array}{c} P_{r}=P_{m}-P_{s}=T_{m}\omega_{r}-T_{e}\omega_{s}=-T_{m\omega s-\omega r\omega s}\omega_{s}=-sT_{m}\omega_{s}=-sP_{s}, \end{array}$$

where “*s*” is defined as the slip of the generator: *s* = (*ω*_*s*_ − *ω*_*r*_)/*ω*_*s*_. The turbine and tracking characteristics are as given in Figure 2.

In this case, power is controlled such that it follows set power-speed characteristics called tracking characteristics. The measurement of actual turbine speed is done and its respective mechanical power is considered as the reference power for the power control loop. In Figure 2, ABCD curve represents the tracking characteristics; reference power is zero from zero to A position, the power between A and B is a straight line, and the speed of B is to be greater than A. In between B and C, the characteristic is the locus of the maximum turbine power. Further, the characteristic is straight line from C to D.

### 2.1. DFIG Modeling [26]

DFIG modeling is carried with an induction machine controlled in a synchronously rotating *dq*-axis frame, with the *d*-axis found to be oriented along the stator-flux vector position. This approach forms stator-flux orientation (SFO) control and is used for modeling DFIG. Following this manner, a decoupled control existing between the electrical torque and the rotor excitation current is noted. As a result, the active and reactive powers are controlled independently of each other. Based on this the stator and rotor voltage equations are given by

(4) $$\begin{array}{c} v_{ds}=R_{s}i_{ds}-\omega_{s}\lambda_{qs}+\frac{d\lambda_{ds}}{dt},\\ v_{qs}=R_{s}i_{qs}+\omega_{s}\lambda_{ds}+\frac{d\lambda_{qs}}{dt},\\ v_{dr}=R_{r}i_{dr}-\left(s\omega_{s}\right)\lambda_{qr}+\frac{d\lambda_{dr}}{dt},\\ v_{qr}=R_{r}i_{qr}+\left(s\omega_{s}\right)\lambda_{dr}+\frac{d\lambda_{qr}}{dt}. \end{array}$$

In the above equations stator voltages and stator currents are given by *v*_*s*_ and *i*_*s*_, rotor voltages and currents are represented as *v*_*r*_ and *i*_*r*_, stator and rotor resistances are given by *R*_*s*_ and *R*_*r*_, and *λ*_*s*_ and *λ*_*r*_ are, respectively, stator and rotor flux linkage components. As the machine is rotating in *dq*-axis frame, the index “*d*” represents direct axis component of the reference frame and “*q*” represent quadrature axis components of the reference frame. In each of the equations (4), the given flux linkages are defined by

(5) $$\begin{array}{c} \lambda_{ds}=-L_{s}i_{ds}+L_{m}i_{dr},\\ \lambda_{qs}=-L_{s}i_{qs}+L_{m}i_{qr},\\ \lambda_{dr}=L_{r}i_{dr}+L_{m}i_{ds},\\ \lambda_{qr}=L_{r}i_{qr}+L_{m}i_{qs}, \end{array}$$

where *L* in each of the cases represent their inductances and *L*_*m*_ is the magnetizing inductance. Equations (4) through (5) represent the modeled equations for the considered DFIG system.

### 2.2. Description of the Conventional DFIG Control System

Numerous papers [20–26] have presented the conventional DFIG control system, which possess two control modules: rotor-side converter (RSC) control system and grid-side converter (GSC) control system. This section discusses the description of the conventional DFIG control system wherein in this module no steps are taken to handle the fault ride-through (FRT) of the DFIG. This conventional control module with RSC and GSC will generally be merged with that of the hardware like STATCOM or crowbar system with the limitations discussed in Section 1. The conventional DFIG control system is presented here in order to make the lucid difference between the applicability of the proposed optimized control module and that of the conventional control system module.

#### 2.2.1. Rotor-Side Converter Control System

**Figure 3:**
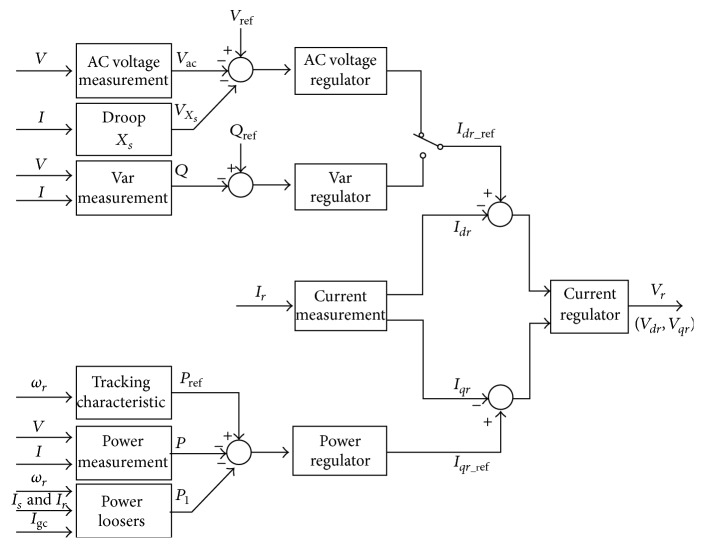
Rotor-side converter control system.

Figure 3 depicts the rotor-side converter control system. The reactive current flowing in the rotor-side converter *C*_ROTOR_ controls the voltage or the reactive power at the grid terminals. The main objective of RSC is to regulate the stator active and reactive power independently. To have decoupled control of the stator active (*P*_*s*_) and reactive power (*Q*_*s*_), the rotor current gets transformed to *d*-*q* components employing the reference frame oriented with that of the stator flux. The *q*-axis current component (*I*_*qr*_) controls the stator active power (*P*_*s*_) and the reference value of the active power (*P*_ref_) as in Figure 3 is obtained using the tracking characteristics via Maximum Power Point tracking. The measured stator active power will get subtracted from the reference value of the active power and the error is driven to the power regulator (power controller module). The output of the power regulator is *I*_*qr*_ref_, which is the reference value of the *q*-axis rotor current. This value will be compared with that of the actual value (*I*_*qr*_) and that error is flowed through the current regulator (current controller), whose output is *v*_*qr*_ (reference voltage for the *q*-axis component).

Until the reactive current is within the maximum current values given by the converter rating, the voltage gets regulated at the reference voltage. On operating the wind turbine in VAR regulation mode, the grid terminals reactive power is maintained constant by a VAR regulator. In this work, a DFIG which fed a weak AC grid is considered; henceforth AC voltage regulator component is used and the reactive controller (VAR regulator) module is not used. The output of the AC voltage regulator is the reference *d*-axis current (*I*_*dr*_ref_), which must be injected in the rotor by the rotor-side converter (*C*_ROTOR_). The same current regulator (current controller) as that of the power regulator is used to regulate the actual (*I*_*dr*_) component of positive-sequence current to its reference value. The output from this regulator is the *d*-axis voltage (*V*_*dr*_) generated by rotor-side converter (*C*_ROTOR_). The current regulator is provided with the feed forward terms that predict *V*_*dr*_. *V*_*dr*_ and *V*_*qr*_ denote the *d*-axis and *q*-axis voltages, respectively. The magnitude of the reference rotor current *I*_*r*_ref_ is given by

(6) $$\begin{array}{c} I_{r\_ref}=I_{dr\_ref}+I_{qr\_ref}. \end{array}$$

The maximum value of this rotor current is limited to 1 pu. On the other hand when *I*_*dr*_ref_ and *I*_*qr*_ref_ are such that their magnitude is higher than 1 pu, then the *I*_*qr*_ref_ component is reduced to bring the magnitude to 1 pu.

#### 2.2.2. Grid-Side Converter Control System

**Figure 4:**
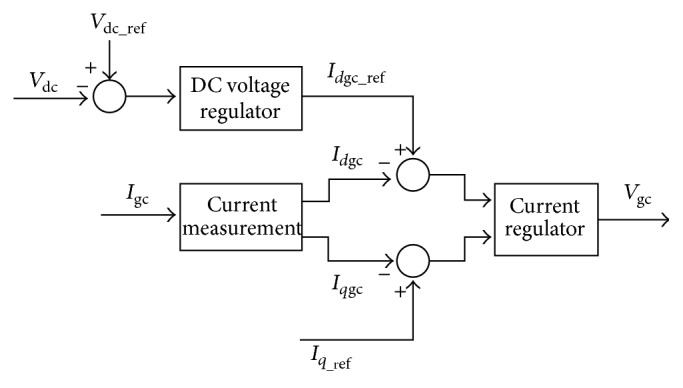
Grid-side converter control system.

Figure 4 shows the schematic of grid-side converter control system. The GSC control system is designed to regulate the voltage of the DC bus capacitor, that is, to maintain the DC-link voltage constant. Also, GSC module is used to generate or absorb reactive power.

GSC control systems consist of a measurement system to measure the *d* and *q* components of AC positive-sequence currents which is to be controlled as well as the DC voltage *V*_dc_. The outer regulation loop consists of a DC voltage regulator. DC voltage regulator output is going to be the reference current (*I*_*d*gc_ref_) to be fed for the current regulator. The current in phase (*I*_*d*gc_) with that of the grid voltage that controls active power flow is also measured and the difference between *I*_*d*gc_ and *I*_*d*gc_ref_ is one of the inputs to the current regulator. A current regulator forms the inner current regulation loop and this regulator controls the magnitude and phase of the voltage generated by *C*_GRID_ from that of *I*_*d*gc_ref_ developed by the DC voltage regulator and given *I*_*q*_ref_. It should be noted that the magnitude of the reference grid converter current is given by

(7) $$\begin{array}{c} I_{gc\_ref}=I_{dgc\_ref}+I_{qr\_ref}. \end{array}$$

The maximum value of this *I*_gc_ref_ is limited to a value defined by the converter maximum power at nominal voltage. When *I*_*d*gc_ref_ and *I*_*q*_ref_ are such that the magnitude is higher than this converter maximum power, then the *I*_*q*_ref_ component is minimized to revert back the magnitude to the maximum value. The DC voltage is controlled with the signal *i*_*d*gc_ref_ and the reactive power is controlled by means of *I*_*q*_ref_ from the reactive power regulator [27–29].

## 3. Description of the Proposed DE-IDWNN Optimized Control System

The conventional DFIG control system discussed in Section 2 does not take into account the fault ride-through of the induction generator. Thus, in this paper, attempt is made to carry out ride-through fault eliminating the extra hardware component. The optimized control module is designed in a manner to accomplish optimal synchronization between the rotor-side converter control system and grid-side converter control system and can handle the disturbances occurring in the system because of the fault. This is taken care of in the system even with the wind turbine feeding a weak AC grid. At this juncture, it should be noted that the proposed controller is to perform effectively within a short span of time and has to be not influenced by the measured noise that might interrupt in the system. This controller is also to be designed to take care of lacking machine parameters information of the system.

All the above discussed conditions will result in added nonlinearity of the system. Hence, to address the said difficulties as well as handle the unavoidable nonlinearity introduced in the system, the proposed DE-IDWNN controller design lay on the evolutionary computational intelligence techniques and will provide a better solution in comparison with that of the conventional approaches. To be more accurate the controller design is performed with a double wavelet neural network controller, whose weights are optimized and tuned employing differential evolution. The controllers employed are IDWNN controllers and the tuning of IDWNN controller meets the fault ride-through. IDWNN-FRT is carried out employing DE. Further, the applicability of DE also performs weight optimization of the neural network controller to achieve better solution and faster convergence.

### 3.1. Differential Evolution Algorithm: An Overview

A population based stochastic evolutionary algorithm introduced by Storn and Price [30] is differential evolution algorithm which is in a way similar to genetic algorithms [31] employing the operators crossover, mutation, and selection and searches the solution space based on the weighted difference between the two population vectors. To differentiate, genetic algorithmic approach relies on crossover and differential evolution approach relies on mutation operation. Mutation operation is used in DE algorithm as a search mechanism and the selection operation directs the search towards the prospective regions of the search space. Initially in DE, populations of solution vectors are generated randomly at the beginning and this initially generated population is improved over generations using mutation, crossover, and selection operators. In the progress of DE algorithm, each of the new solutions which resulted competes with that of the mutant vector and the better ones in the race win the competition. The standard DE is as given in Pseudocode 1.

### 3.2. Proposed Inertia Based Double Wavelet Neural Network

**Figure 5:**
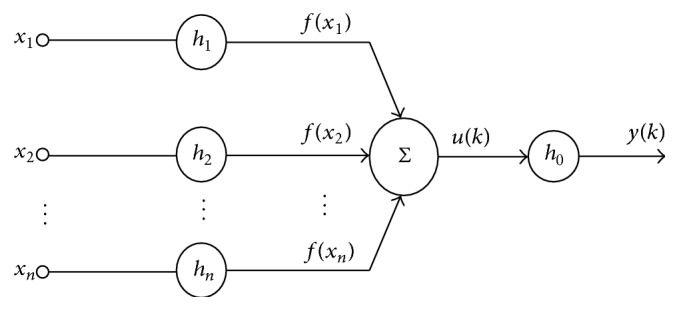
Architecture of inertia based double wavelet neural network.

The neural network architecture of the proposed inertia based double wavelet neural network is shown in Figure 5. It consists of input layer, hidden layer 1, hidden layer 2, and the output layer. Hidden layer 1 contains *n*-wavelet synapses with *h*_1_ wavelet functions and hidden layer 2 contains one wavelet synapse with *h*_2_ wavelet functions.

Vector signal is as follows:

(8) $$\begin{array}{c} x\left(k\right)=\left(x_{1}\left(k\right),x_{2}\left(k\right),\ldots ,x_{n}\left(k\right)\right), \end{array}$$

where *k* = 0,1, 2,…, *n* which is the number of sample inputs in the training set.

The output of the proposed inertia based double wavelet neural network is expressed as

(9) $$\begin{array}{c} y\left(k\right)=f\left(\overset{n}{\underset{i=1}{\sum }}f\left(x_{i}\left(k\right)\right)\right)=f\left(u\left(k\right)\right)\\ =\overset{h_{2}}{\underset{q=0}{\sum }}\varphi_{q0}\left(\overset{n}{\underset{i=1}{\sum }}\;\overset{h_{1}}{\underset{j=0}{\sum }}\varphi_{ji}\left(x_{i}\left(k\right)\right)w_{ji}\left(k\right)\right)w_{j0}\\ y\left(k\right)=\overset{h_{2}}{\underset{q=0}{\sum }}\varphi_{20}\left(u\left(k\right)w_{q0}\left(k\right)\right), \end{array}$$

where *w*_*ji*_(*k*) represents synaptic weights.

**Figure 6:**
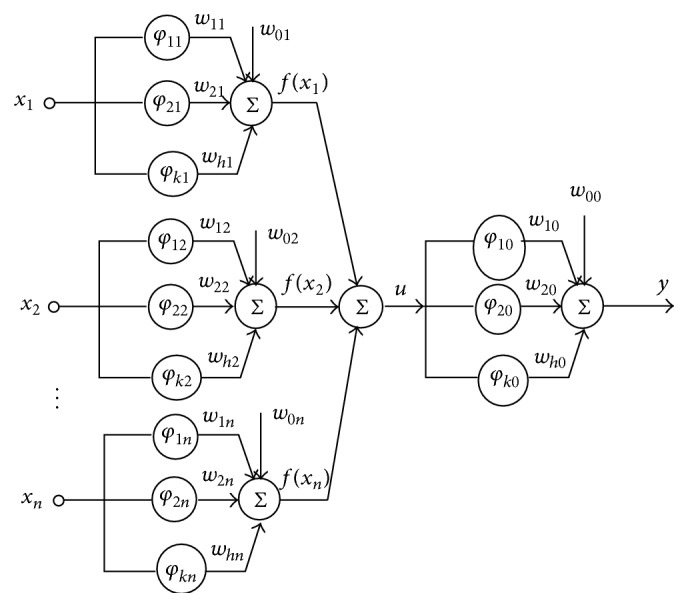
Architecture of proposed IDWNN with nonlinear wavelet synapse.

The wavelets differ between each other by dilation; translation and bias factors are realized in each wavelet synapse. The architecture of proposed inertia based wavelet neural network with nonlinear wavelet synapses is shown in Figure 6.

The expression for tuning of the output layer is given by

(10) $$\begin{array}{c} E\left(k\right)=\frac{1}{2}{\left(d\left(k\right)-y\left(k\right)\right)}^{2}=\frac{1}{2}e^{2}\left(k\right), \end{array}$$

where *d*(*k*) is external training signal. The learning algorithm of output layer of the proposed neural network is

(11) $$\begin{array}{c} w_{j0}\left(k+1\right)=\eta w_{j0}\left(k\right)+\psi_{0}\left(k\right)e\left(k\right)\varphi_{j0}\left(u\left(k\right)\right). \end{array}$$

Equation (11) can be represented in vector form as

(12) $$\begin{array}{c} w_{0}\left(k+1\right)=\eta w_{0}\left(k\right)+\psi_{0}\left(k\right)e\left(k\right)\varphi_{0}\left(u\left(k\right)\right), \end{array}$$

where *e*(*k*) is learning error, *ψ*_0_(*k*) is learning rate parameter, and *η* is inertia factor.

The analogy of the learning algorithm is given by

(13) $$\begin{array}{c} w_{0}\left(k+1\right)=w_{0}\left(k\right)+\frac{e\left(k\right)\varphi_{0}\left(u\left(k\right)\right)}{\gamma_{i}^{w_{0}}\left(k\right)},\\ \gamma_{i}^{w_{0}}\left(k+1\right)=\alpha \gamma_{i}^{w_{0}}\left(k\right)+{\left\|\varphi_{0}\left(u\left(k+1\right)\right)\right\|}^{2}. \end{array}$$

The range of *α* is between 0 and 1. The expression for tuning of the hidden layer is

(14) $$\begin{array}{c} E\left(k\right)=\frac{1}{2}{\left(d\left(k\right)-f_{0}\left(u\left(k\right)\right)\right)}^{2}=\frac{1}{2}{\left(d\left(k\right)-f_{0}\left(\overset{n}{\underset{i=1}{\sum }}\overset{h_{2}}{\underset{j=0}{\sum }}\varphi_{ji}\left(x_{i}\left(k\right)\right)w_{ji}\left(k\right)\right)\right)}^{2}. \end{array}$$

Learning algorithm of hidden layer of the proposed neural network is

(15) $$\begin{array}{c} w_{ji}\left(k+1\right)=\eta w_{ji}\left(k\right)+\psi \left(k\right)e\left(k\right)f_{0}^{\prime }\left(u\left(k\right)\right)\varphi_{ji}\left(x_{i}\left(k\right)\right). \end{array}$$

Equation (15) can be represented in vector form as

(16) $$\begin{array}{c} w_{i}\left(k+1\right)=\eta w_{i}\left(k\right)+\psi \left(k\right)e\left(k\right)f_{0}^{\prime }\left(u\left(k\right)\right)\varphi_{i}\left(x_{i}\left(k\right)\right), \end{array}$$

where *e*(*k*) is learning error, *ψ*(*k*) is learning rate parameter, and *η* is inertia factor.

The analogy of the learning algorithm in (15) is given by

(17) $$\begin{array}{c} w_{i}\left(k+1\right)=w_{i}\left(k\right)+\frac{e\left(k\right)f_{0}^{\prime }\left(u\left(k\right)\right)\varphi_{i}\left(x_{i}\left(k\right)\right)}{\gamma_{i}^{w_{i}}\left(k\right)},\\ \gamma_{i}^{w_{i}}\left(k+1\right)=\alpha \gamma_{i}^{w_{i}}\left(k\right)+{\left\|\varphi_{i}\left(x_{i}\left(k+1\right)\right)\right\|}^{2}. \end{array}$$

The range of *α* is between 0 and 1. The properties of this proposed algorithm have both smoothing and approximating. The inertia parameter helps in increasing the speed of convergence of the system. The inertia parameter also prevents the network from getting converged to local minima. The inertia factor is between values 0 and 4.

### 3.3. Proposed DE Based IDWNN Optimization Controller

Based on the given inputs and outputs of the system, the proposed inertia based double wavelet neural network model is designed and the DE approach is employed to tune the weights of the IDWNN and to tune the outputs of the neural network model. The proposed pseudocode for DE based IDWNN optimization controller is as given in Pseudocode 2.

The proposed DE-IDWNN controller is applied to handle fault ride-through of grid-connected doubly fed induction generators and the methodology adopted for handling fault ride-through using this proposed controller is presented in the following section.

## 4. Fault Ride-Through of DFIG Module Using Proposed DE-IDWNN Controller

**Figure 7:**
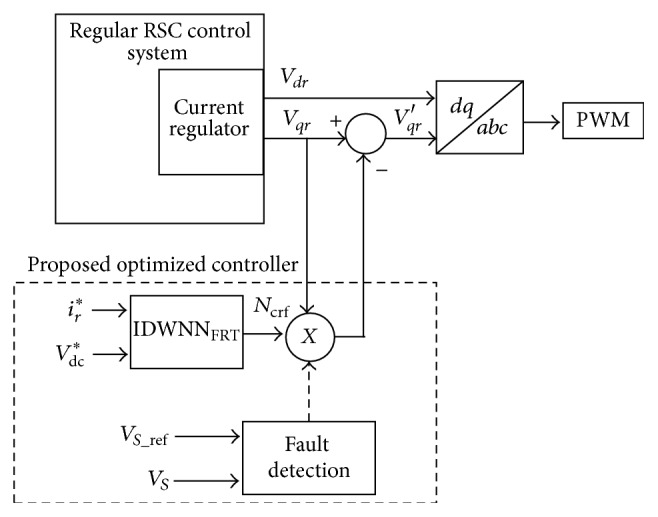
Proposed DE-IDWNN optimized control system.

DFIG module with fault ride-through employing proposed DE-IDWNN controller is as shown in Figure 7. In the proposed design, GSC control system remains the same as that of the conventional DFIG module and the modification occurs in the RSC control system module.

The proposed optimized controller starts its operation only when the AC voltage *v*_*s*_ exceeds ten percent higher than that of the set reference voltage. The constraints that should be taken care of for safe guarding the DFIG are the DC-link overvoltage and the rotor overcurrent. Both these constraints should not exceed their set limits in the considered restoring period. Also, care should be taken in order to transfer the additional energy generated in the rotor via the converters to the grid. This process of transferring the additional energy induced will enable the DC-link voltage and rotor current to maintain their respective normal values. A setback on performing this operation is that when the rotor current is decreased by fast transfer of the stored energy from rotor to grid, there might be a chance that the DC-link voltage increases abruptly and it may get deviated from the normal limits. On the other hand, without fast mechanism, slowly decreasing the rotor current such that the DC-link voltage does not exceed the limit may result in the rotor current reaching abnormal transient points. Henceforth, for implementing an effective and efficient fault ride-through, the transition signals of the rotor current should also consider the nominal values of the DC-link voltage. The proposed controller is the inertia double wavelet neural network controller and the input to IDWNN_FRT_ is *V*_dc_^*∗*^ and *i*_*r*_^*∗*^ and the output of the neural network controller is *N*_crf_. *V*_dc_^*∗*^ and *i*_*r*_^*∗*^ act as the inputs to the IDWNN optimized controller and are given by

(18) $$\begin{array}{c} V_{dc}^{*}=\frac{V_{dc}-V_{ss}}{V_{max}-V_{ss}},\\ i_{r}^{*}=\frac{i_{r}-i_{ss}}{i_{max}-i_{ss}}, \end{array}$$

where *V*_ss_ and *i*_ss_ indicate the steady state values, *V*_max_ and *i*_max_ specify the maximum values, *i*_*r*_ is the rotor rms current, and *V*_dc_ is the DC-link voltage.

Both the input values are normalized before they are fed into the neural network controller. Design of neural network controller is performed as shown in Table 1 and the training process of the controller is carried out to obtain *N*_crf_. Originally the controller is designed by considering random weights into the IDWNN and then DE is adopted for tuning the weight values of the IDWNN controller. Nonlinear wavelet synapse is employed during the training process for faster convergence.

Along with the proposed IDWNN controller, differential evolution is employed to tune the weights of IDWNN controller as well as minimize the objective function or the fitness function as given in

(19) $$\begin{array}{c} \min f={\left[\frac{V_{dc\;max}-V_{ss}}{V_{max}-V_{ss}}\right]}^{2}+{\left[\frac{i_{rmax}-i_{ss}}{i_{max}-i_{ss}}\right]}^{2}. \end{array}$$

*V*_max_ and *i*_*r*max_ represent the maximum value of the signals during the entire restoring duration. On applying DE for the considered problem, each set of variables is represented by binary strings called chromosomes and chromosomes made up individual entities called genes. All the chromosomes generated result in the formation of population. In this problem under consideration, population size is 20, that is, 20 chromosomes, and the length of chromosome is taken to be 8; that is, 8 genes comprise a chromosome. The initial 6 genes represent the binary strings of the range of input parameters and the remaining genes represent the output range. The process of DE is carried out as given by the pseudocode in Pseudocode 1. DE is invoked and activated to search for minimizing the optimization function given in (19). In DE process, mutation is carried out at the initial process and then crossover and selection operations are performed. The search process is carried out to find the maximized value for (19) and the procedure is continued until a specified stopping condition is reached.

**Figure 8:**
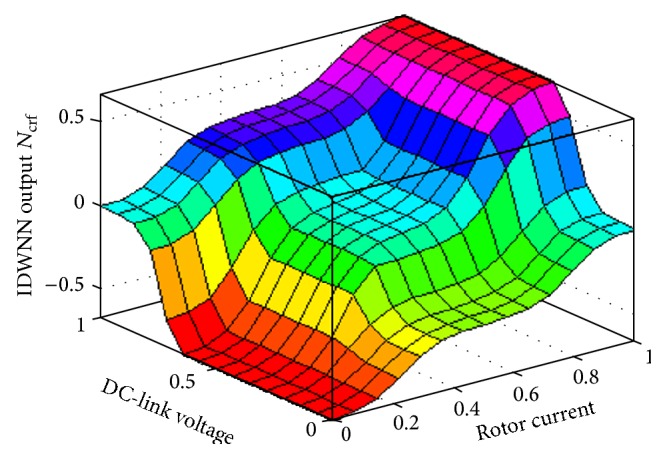
Three-dimensional output for the trained controller.

The importance of the fitness function in (19) is as follows: generally, for a variety of problems, the objective function will be an integral function where the output is the complete behavior of the system in a particular time interval. In this problem domain, the aim is to limit the instantaneous value of rotor current and DC-link voltage so as to eliminate the tripping of DFIG. As a result, the objective function is the sum of the squared components that are to be minimized. Also, it is to be noted, in this case, that the aim here is not only to minimize the specified objective function in (19), but also to maintain the balance between the rotor current and DC-link voltage in the range of acceptable values.  Figure 8 shows the three-dimensional graph for the IDWNN_FRT_ output *N*_crf_ with respect to *V*_dc_^*∗*^ and *i*_*r*_^*∗*^ after the DE optimization. The proposed DE-IDWNN controller can be employed for various sizes of machines. The training of the neural network controller and the DE optimization process remains the same in all cases.

## 5. Numerical Experimentation and Simulation Results

**Figure 9:**
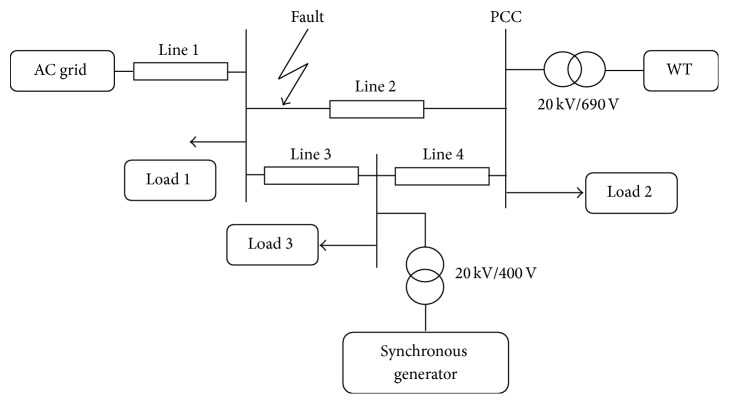
Electrical system considered for validating the proposed controller.

The proposed DE-IDWNN controller is validated by applying the said control strategy for 1.5 MW wind turbines connected to a 25 kV distribution system exporting power to a 120 kV grid through a 30 km 25 kV feeder. The electrical system [26] considered for validating the proposed control design is given in Figure 9. Also, this work basically deals with handling three-phase symmetrical grid faults. The occurrence of three-phase faults is noted at 0.5 seconds and the simulation is carried out. The simulation response is noted for both the traditional DFIG control system and that of the proposed control system design. From the simulation response of the traditional control system it can be noted that this system requires an auxiliary system for fault ride-through option and observes the limitations as discussed in Section 1. During the process of simulation it is observed that the DFIG along with the proposed controller handles the complete response at fault periods and after fault periods and gets ride-through fault eliminating the applicability of auxiliary hardware.  Table 2 shows the specifications for the parameters of the DFIG system and the grid system [26] considered for simulation. The entire proposed control strategy was run in MATLABR2009 environment and executed in Intel Core2 Duo Processor with 2.27 GHz speed and 2.00 GB RAM. Simulink environment in MATLAB is used to model the converter modules. All the simulations are carried out for wind speed of 12 m/s.

**Figure 10:**
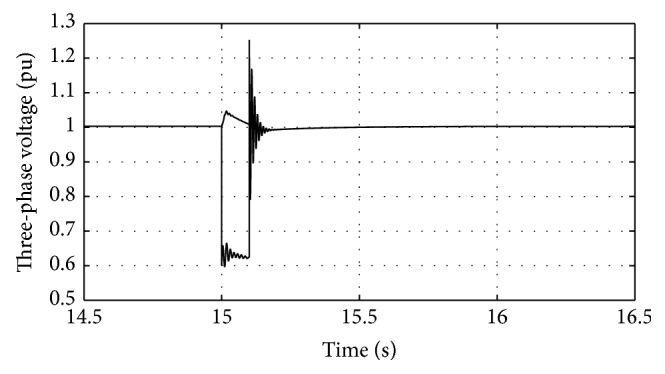
Response of three-phase voltage for traditional control system.

**Figure 11 fig11:**
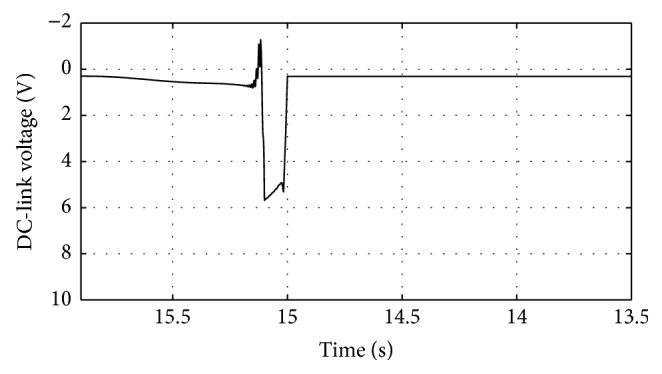
Response of DC-link voltage for traditional control system.

**Figure 12 fig12:**
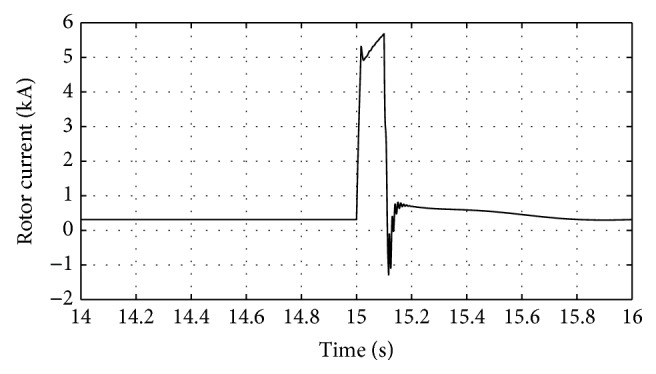
Response of rotor current for traditional control system.

**Figure 13 fig13:**
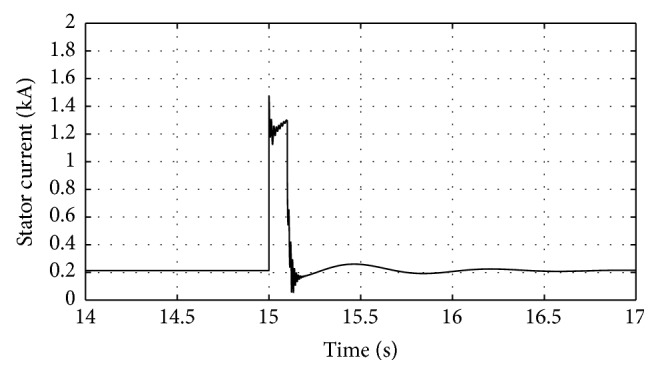
Response of stator current for traditional control system.

**Figure 14 fig14:**
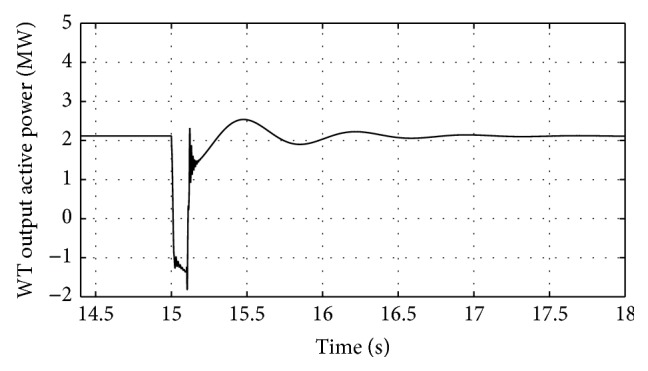
Response of wind turbine active power output for traditional control system.

**Figure 15 fig15:**
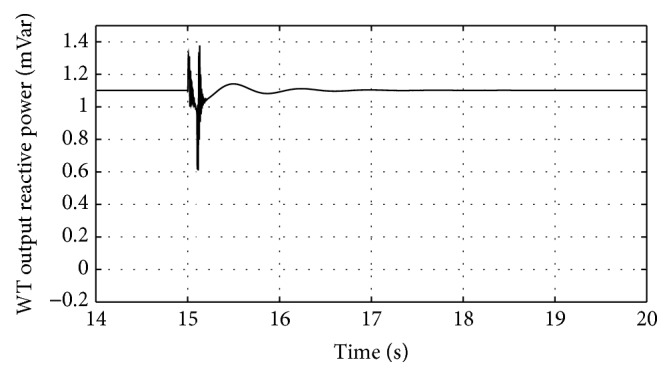
Response of wind turbine reactive power output for traditional control system.

**Figure 16 fig16:**
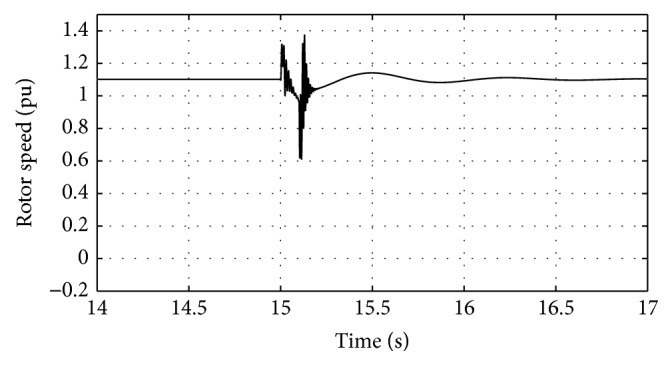
Response of rotor speed for traditional control system.

**Figure 17:**
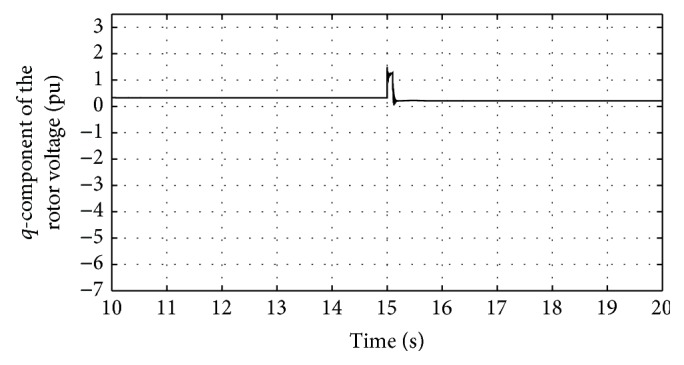
Response of *q*-component of rotor voltage for traditional control system.

**Figure 18:**
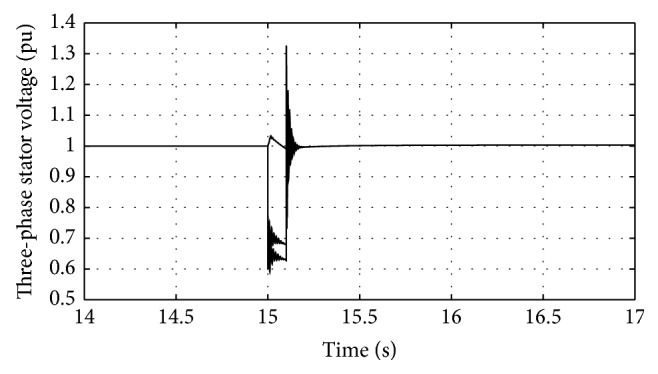
Response of three-phase stator voltage for proposed DE-IDWNN controller.

**Figure 19:**
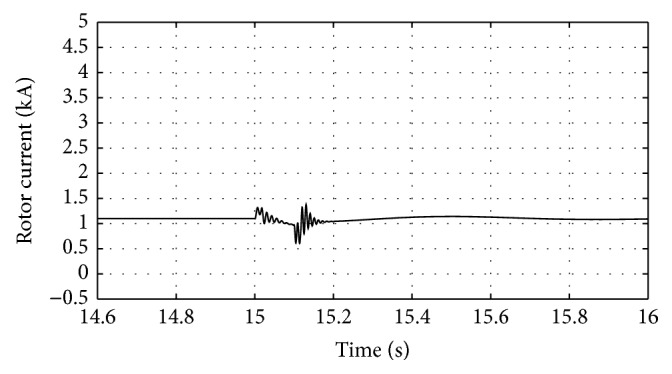
Response of rotor current for proposed DE-IDWNN controller.

**Figure 20:**
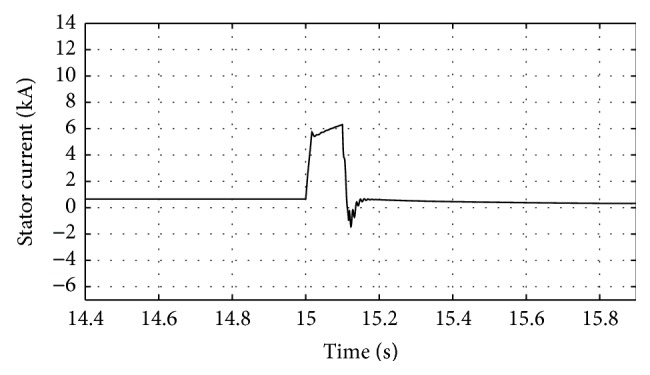
Response of stator current for proposed DE-IDWNN controller.

Figures 10 [Fig fig11] [Fig fig12] [Fig fig13] [Fig fig14] [Fig fig15] [Fig fig16]–17 show the response of the traditional control system generated for the wind speed of 12 m/s. From Figures 10 and 11, it can be observed that the DC voltage exceeds the set limit resulting in damaging the capacitor present. Also, the rotor current increases in an advert manner, nearly 100% compared to that of the acceptable value in the rotor-side converter control system. During this conventional controller action, it is noted that the entire response of the system varies in an erroneous manner resulting in more fluctuations in the grid side. Thus this conventional control design is modified to enable appropriate handling of ride-through fault conditions. The proposed DE-IDWNN controller is simulated for 100 generations and the entire response observed during the simulation response is as shown in Figure 18 through Figure 25. Nonlinear wavelet synapse is utilized for the updating process of IDWNN controller and the DE is initiated to tune the weights of IDWNN controller and that of the objective function as given in (19). The evaluation of the fitness function is studied at 85% voltage dip. The response of the observed parameters during simulation process proves the removal of fluctuations occurring during the conventional control design and as well it is noted that it reaches the steady state at the earliest. The fault ride-through of the DFIG module is achieved at the point wherein optimal solution is arrived at employing the proposed controller design. At this juncture, overvoltages noted at DC-link point and overcurrents at the rotor side are observed to be well within the limit of the maximum set threshold values. As a result, the damaging of the capacitor is protected and fault and postfault occurrence to the grid are controlled in an effective manner. Figures 19 and 20 show the rotor current and stator current with fluctuations reduced obtaining the steady state value at a faster rate.

**Figure 21:**
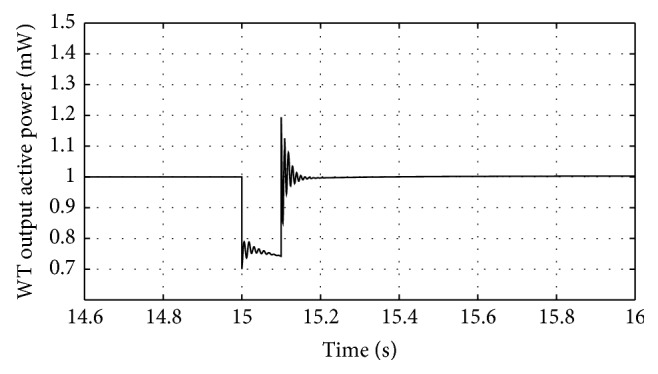
Response of wind turbine output active power for proposed DE-IDWNN controller.

**Figure 22:**
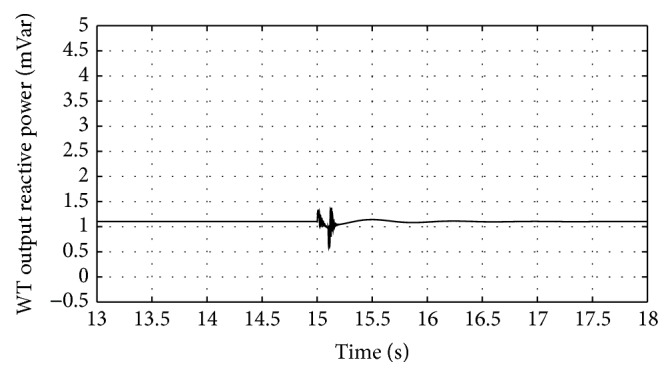
Response of wind turbine output reactive power for proposed DE-IDWNN controller.

**Figure 23:**
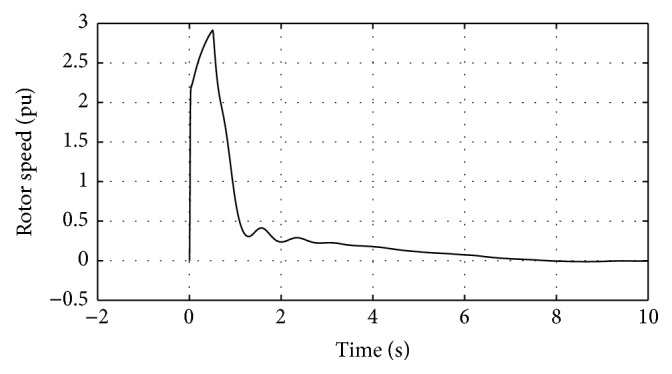
Response of rotor speed for proposed DE-IDWNN controller.

Figures 21 and 22 show the response of the wind turbine output active and reactive power. Fundamentally, in this proposed controller design, RSC will not be cut during the fault condition and this RSC provides the required reactive power to the grid, in case of handling the sufficient voltage drops occurring. But the proposed DE-IDWNN controller acts to the fault effectively arriving at an optimal solution and thus it will prevent more amount of reactive power from being transferred from DFIG after the fault. In the proposed design, DFIG supplies the grid with the required amount of reactive power and is found to sustain that of the grid voltage. At the time of fault, the rotor speed of the proposed controller increases which is seen in Figure 23. The increase in speed of the rotor is noted due to the capacity of the wind turbine to store huge energy. At the occurrence of fault, there will be a huge drop in the voltage and the wind power is transferred as kinetic energy to the rotor without dissipating that of the grid. At the end of the fault duration, the grid receives this energy and the increase in the speed of the rotor will automatically get settled to the earlier value (i.e., the value before the fault has occurred).

**Figure 24:**
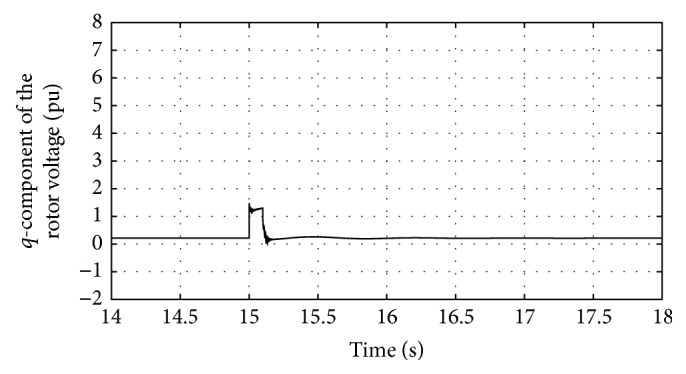
Response of *q*-component of rotor voltage for proposed DE-IDWNN controller.

**Figure 25:**
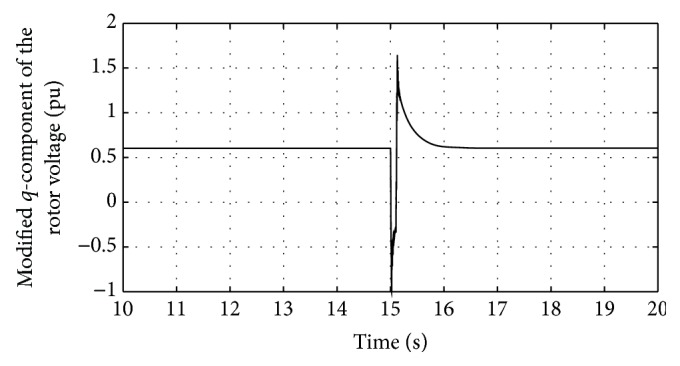
Response of the modified *q*-component of rotor voltage for proposed DE-IDWNN controller.

Figures 24 and 25 show the *q*-component and the modified *q*-component of the rotor voltage of the proposed controller design. The occurrence of transients is noted and the proposed controller acts in a manner to bring back the AC voltage to its set value resulting in the above said transient. In case during simulation process higher voltage dip occurs, DFIG is allowed to disconnect. This is the bad condition on getting connected to the grid. Thus the entire simulation study is carried out for wind speed at 12 m/s and voltage dip of 85%. The proposed controller found that ride-through fault occurred.  Table 3 shows the fitness function values evolved during various generations. At the end of the 100 generations, it is noted that the fitness value reached 4.0021. The DC-link voltage and the rotor current are found to be maintained within the said permissible limits at this optimal point.

**Figure 26:**
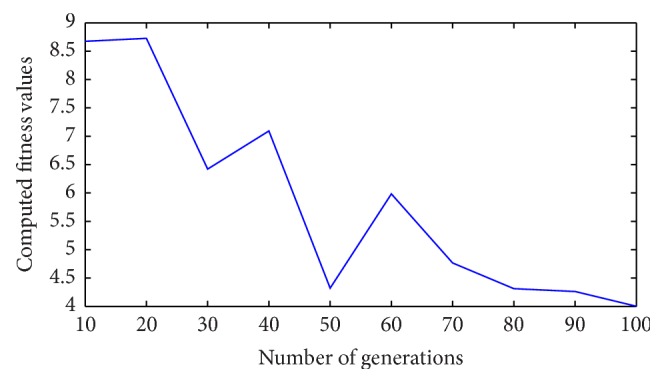
Variation of fitness functions with respect to number of generations.

From Table 4, it can be observed that the proposed controller achieves a minimal fitness function value of 4.0021 in comparison with that of the earlier other methods available in the literature proving the effectiveness of the controller.  Figure 26 shows the variation of fitness function with respect to generations and from Figure 26 it can be inferred that the search process enables the fitness function to reach a minimum value.

## 6. Conclusion

This paper proposed a novel heuristic based controller module employing differential evolution and neural network architecture to improve the low-voltage ride-through rate of grid-connected wind turbines, which are connected along with doubly fed induction generators (DFIGs). The limitation of the traditional control system is taken care of in this work by introducing heuristic controllers that remove the usage of crowbar and ensure that wind turbines supply necessary reactive power to the grid during faults. The controller designed in this paper enhances the DFIG converter during the grid fault and this controller takes care of the ride-through fault without employing any other hardware modules. The proposed controller design is validated for a case study of wind farm with 1.5 MW wind turbines connected to a 25 kV distribution system exporting power to a 120 kV grid. The results simulated prove the effectiveness of the controller design in comparison with that of the methods available in the literature.

## Figures and Tables

**Pseudocode 1:**
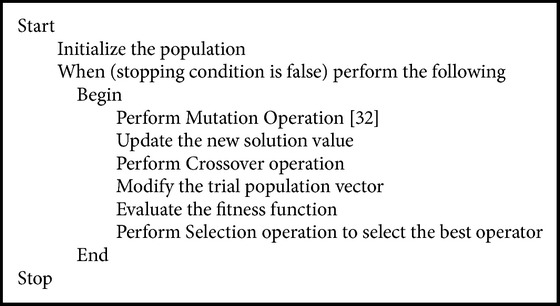
Pseudocode of standard DE.

**Pseudocode 2:**
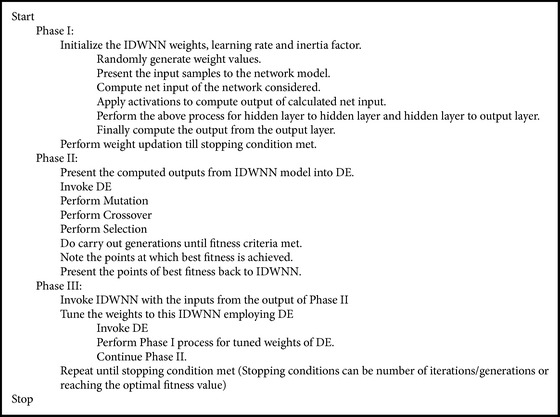
Pseudocode for DE-IDWNN controller.

**Table 1:** Proposed IDWNN controller parameters.

Parameters of the proposed IDWNN controller	Set values
Number of input neurons	2
Number of output neurons	1
Inertia factor	1.75
Number of hidden layer neurons	8
Learning rate parameter	1

**Table 2:** System specifications of DFIG system and grid module.

Specifications of DFIG system	
Rated power	1.5 MW
Rated stator voltage	690 V
Nominal wind speed	12 m/s
Stator resistance	0.00706 pu
Rotor resistance	0.005 pu
Stator leakage inductance	0.1716 pu
Rotor leakage inductance	0.156 pu
Magnetizing inductance	2.9 pu
Turns ratio	2.7
Rotational inertia	5.04 s
Nominal DC-link rated voltage	1200 V
DC bus capacitor	60 millifarads

Specifications of AC grid	

Rated voltage	20 kV
Frequency	50 Hz
Short circuit ratio	2.23
Load 1 power	400 kW & 120 kVar
Load 2 power	500 kW & 150 kVar
Load 3 power	50 KW & 15 kVar

Length of transmission lines	

Line 1 and Line 3	15 km
Line 2 and Line 4	30 km
Synchronous machine speed	1500 rpm
Synchronous machine power	85 kVA

**Table 3:** Fitness function evolved during generations.

Generations	Fitness function (*f*) as in (19)
10	8.6741
20	8.7259
30	6.4213
40	7.0932
50	4.3218
60	5.9831
70	4.7651
80	4.3124
90	4.2618
100	4.0021

**Table 4:** Comparison of the fitness function values.

Methods employed	Optimal best value of “*f*” in (19)
GA based approach [26]	9.5612
ANN controller [5]	7.2314
ANFIS controller [20]	7.1230
Proposed DE-IDWNN controller	4.0021
